# Measuring the risk factors for postpartum depression: development of the Japanese version of the Postpartum Depression Predictors Inventory-Revised (PDPI-R-J)

**DOI:** 10.1186/1471-2393-13-112

**Published:** 2013-05-14

**Authors:** Mari Ikeda, Kiyoko Kamibeppu

**Affiliations:** 1 Department of Family Nursing, Division of Health Science & Nursing, Graduate School of Medicine, The University of Tokyo, 7-3-1 Hongo Bunkyo, Tokyo 1130033, Japan

**Keywords:** Japanese, Postpartum depression, Risk factors, ROC analysis, Screening instrument

## Abstract

**Background:**

Postpartum depression (PPD) is a global phenomenon. Depression in the first month following delivery is experienced by 20% of mothers in Japan. Therefore, a screening instrument that identifies the risk for depression during pregnancy and in the early postpartum period is required for primary prevention. The aims of this study were to develop the Japanese version of the Postpartum Depression Predictors Inventory-Revised (PDPI-R-J) and determine its predictive validity during pregnancy and one month after delivery.

**Methods:**

In order to develop the inventory, two bilingual translators translated the PDPI-R into Japanese. Then, back translation was done and a thorough discussion with the original developer was conducted in order to establish semantic equivalence. After the PDPI-R-J was developed, the study used a prospective cohort design. A total of 84 women in their eighth month of pregnancy participated in the study. Seventy-six mothers completed the PDPI-R-J at the first month after childbirth. Women were diagnosed using Mini-International Neuropsychiatric Interview (M.I.N.I.) to determine the presence of minor or major depression at the first month after childbirth and the receiver operating characteristic curve was plotted to evaluate the predictive capacity of PDPI-R-J.

**Results:**

Of the 76 mothers who completed the PDPI-R-J during the first-month assessment, 16 mothers (21%) met the PPD criteria. The prenatal version of the PDPI-R-J administered during pregnancy accurately predicted 62.8% of PPD (95% CI 0.48–0.77) and the postpartum version administered at the first month after delivery predicted 82.0% of PPD (95% CI 0.71–0.93). The cutoffs identified were 5.5 for the prenatal version and 7.5 for the postpartum version. The PDPI-R-J postpartum version, which includes items relating to the infant, increased the predictive validity of PPD (0.67 to 0.82). Comments from the participants included that the use of the PDPI-R-J enhanced the chance to openly communicate about their history and risks for depression with the researchers, if any existed.

**Conclusions:**

The PDPI-R-J was found to be a useful and valid screening tool for predicting PPD. Both the prenatal and postpartum versions should be continuously administered to mothers because delivery and infant-related factors affect the potential for PPD.

## Background

Despite the stereotype that the perinatal phase is a period of contentment, women frequently experience adjustment difficulties and depressive symptoms during pregnancy and the postpartum period. Approximately 8.5%–11% of women experience either minor or major depression (mMD) during pregnancy. Furthermore, 19% of women have mMD in the first 3 months following delivery [1]. A recent large-scale epidemiological study provided some evidence of increased risk for depression in the postpartum period compared with non-pregnant/non-postpartum women (adjusted odds ratio: 1.52; 95% CI: 1.07–2.15) [2]. In Japan, depression is reported to occur in the first month following delivery in 20.4% of mothers [3].

Women experiencing postpartum depression (PPD) appear to be unhappy, irritable, and unable to cope; have negative feelings about themselves and their children; are anxious; have low libido; have marital problems; experience difficulties managing household tasks; are tearful; have physical symptoms, such as sleep and appetite disturbances; and display obsessional behavior [4]. The quality of life for such women and their families is severely compromised, which can result in marital breakdown [5]. In the most severe cases, women report fear of hurting themselves or their newborns [6].

Risk factors for PPD include previous depression, anxiety and depressive symptoms during pregnancy, stressful recent life events, lack of social support, and low self-esteem [7,8]. It is crucial that early and accurate identification and intervention occur in order to prevent long-term consequences for childbearing families and to prevent PPD from becoming a significant mental-health problem. A screening instrument that identifies the risk for depression during pregnancy and in the postpartum period would help to prevent aggravation of PPD even when it occurs. Since 1978, when the first checklist to identify the risk factors for PPD in pregnancy became available [9], several similar instruments have been developed. Currently, 12 available instruments are designed to assess the risk factors for PPD (Table 1). Most such instruments include items like histories of depression and social support [10-17]. The instruments that have been developed since the late 1990s include self-esteem items [14,15,17,18]. Boyce et al. (2001) developed the Vulnerable Personality Style Questionnaire (VPSQ) to reflect dimensions of personality that are associated with vulnerability to PPD [19]. The instrument by Webster et al. (2003) [16] combined prenatal, perinatal, and postnatal risk factors for PPD; however, this instrument is used in the postpartum period, not during pregnancy.

**Table 1:** Comparison of the PDPI-R with previous screening instruments

**Beck, 2001**	***Braverman *et al.*, 1978***	***Petrick, 1987***	***Boyer *et al.*, 1990***	***Appleby *et al.*, 1994***	***Stamp *et al.*, 1996***	***Posner *et al.*, 1997***	***Reid *et al.*, 1998***	***Boyce *et al.*, 2001***	***Webster *et al.*, 2003***	***Austin *et al.*, 2005***	***Bernazzani *et al.*, 2005***
**PDPI-R**	*	*	*	** ASQ**	**MASQ**	** AQ**	**ALPHA**	** VPSQ**	** VPDI**	** PRQ**	** CAME**
Marital status	X										
Socioeconomic status			X			X					
Self-esteem						X	X			X	X
Prenatal depression			X	X		X	X			X	
Prenatal anxiety		X	X	X		X		X		X	
Unwanted/unplanned pregnancy	X	X	X	X			X			X	X
Depression history	X	X	X	X	X	X	X		X	X	
Social support		X	X	X	X	X	X		X	X	X
Marital relationship/satisfaction	X		X	X	X	X	X			X	X
Life stress		X	X	X			X			X	X
Childcare stress											
Infant temperament									X		
Maternity blues						X			X		

*no specific name was given to the instrument.

The Postpartum Depression Predictors Inventory-Revised (PDPI-R) [20] is the only antenatal screening scale whose design has been based on the findings of meta-analyses [21,22] and not solely on clinical experience. The PDPI-R was updated to allow the identification of further risk factors [23], and it was the first instrument to assess risk factors that occur during both pregnancy and the postpartum period. The PDPI-R was originally designed to be administered via an interview conducted by a clinician. The interview format provides a woman with an opportunity to discuss any problems she may be experiencing in relation to the risk factors [24]. The PDPI-R was adapted to enable women to complete the checklist themselves [25]. In that study, midwives were enthusiastic about the use of the PDPI-R, because pregnant women were given a chance to consider their responses before their perinatal consultation, and they appeared eager to explain them. Women were also pleased to have the opportunity to discuss their feelings and to consider their situations as being a normal occurrence. Thus, the PDPI-R enhanced communication between clinicians and women in need by focusing on their responses to the checklist questions.

The recent format of the PDPI-R is a self-report version with some follow-up questions. For example, if a woman has felt depressed during her current pregnancy, 1 point is assigned. The two follow-up questions (when/how long, how severe), which are not scored, are asked if a woman answers that she has felt depressed during pregnancy. The purpose of knowing the timing, duration, and severity of depression is to provide clinicians with additional information to help them determine whether a woman needs a mental-health referral. The follow-up questions facilitate dialogue between women and clinicians. The original developer of the PDPI-R provided scoring directions for both the prenatal version and the postpartum version [24] and evaluated the psychometric properties of the PDPI-R [6]. The prenatal PDPI-R yielded a sensitivity of 0.76 and a specificity of 0.54 at a cutoff score of 10.5 (Beck et al. 2006). The predictive validity of the PDPI-R a screening instrument for PPD was determined in a large sample of Italian women [26]. The prenatal version of the PDPI-R administered during the 8^th^ month of pregnancy and the postpartum version administered during the 1^st^ month after delivery accurately predicted 78.2% and 83.4% of PPD, respectively. The cutoffs identified were 3.5 for the prenatal version and 5.5 for the postpartum version. The authors concluded that the PDPI-R is a useful and valid screening tool for PPD. The PDPI-R assesses individual risk factors, and the total score could be used to screen for PPD.

Our previous research has revealed evidence that in Japan, only 15% of hospitals perform maternal depression screening during the perinatal period; furthermore, only 9% of Japanese hospitals administer depression screening during the antenatal period [27]. In contrast, in the United States, 47% of women were screened for maternal depression during their prenatal visits [28]. Japanese women are advised to have prenatal visits every 4 weeks through 23 weeks gestation (instead of 27 weeks, as in the United States), every 2 weeks at 24–35 weeks gestation, and weekly after 36 weeks gestation for a total of 14 prenatal visits per low–medical-risk pregnancy [29]. In each prenatal visit, an appointment with the midwife follows the consultation with the obstetrician. It would be useful to utilize the frequent contact with women during the perinatal period to implement an adequate screening instrument that identifies the risk for depression during pregnancy; this would be a useful step in the primary prevention of PPD. The aims of this study were to develop a Japanese version of the PDPI-R (PDPI-R-J), to assess its reliability, and to evaluate its sensitivity, specificity, and predictive value as a screening instrument for PPD as compared with those of the PDPI-R over a range of cutoff scores.

## Methods

### Setting and participants

The setting for this study was a university hospital. All primipara women who were planning to give birth at the university hospital were potentially eligible for the study. In the first phase of recruitment, we explained the aims of the study and asked women who were present at the antenatal class (Mother Class) if they were willing to participate. The data were collected from October 2009 to September 2010. Out of 135 eligible women, 91 gave written informed consent (67.4%). Women who agreed to participate handed in application forms that they completed on site. Women who agreed received either an e-mail or a phone call from the first author (M.I.; the researcher) to schedule the baseline assessment. To be included in the study, a woman had to be in the 28^th^–32^nd^ gestational weeks, married, carrying a single fetus, willing to sign an informed consent release form, and available to be contacted by either phone or e-mail. Exclusion criteria for the study were; age <20 years, multiparous, difficulty speaking Japanese, and judged by midwives/obstetricians to be at risk of mental instability. We were able to collect information only after a signed informed consent form was received; therefore, the sociodemographic characteristics of women who did not participate in the study were not available.

### Procedure and design

This study used a prospective cohort design. The study began in the participant’s 8^th^ month of pregnancy and ended at 1 month after childbirth. The prenatal version of the PDPI-R-J was administered at the 8^th^ month of pregnancy and the postpartum version at the 1^st^ month after childbirth. In each assessment, a short interview regarding each risk factor and follow-up questions was followed by the first author (MI). The Mini-International Neuropsychiatric Interview (M.I.N.I.) was administered by a trained psychologist in order to determine whether the subject met the DSM-IV criteria for mMD (the outcome measure) at 1 month after childbirth.

### Risk factors assessment

The 13-item original English version of the PDPI-R was translated into Japanese and then back-translated into English in order to reach semantic equivalence. The 13 PDPI-R factors included: (1) marital status (being single), (2) low socioeconomic status, (3) low self-esteem, (4) prenatal depression, (5) prenatal anxiety, (6) unintentional pregnancy, (7) prior depression, (8) lack of social support, (9) marital dissatisfaction, (10) life stress, (11) childcare stress, (12) infant temperament, and (13) maternity blues. The first ten predictors comprise the prenatal version of the PDPI-R, whereas the last three risk factors are specific to the postpartum period. Total scores on the prenatal version of the PDPI-R range 0–32. The PDPI-R postpartum version (prenatal plus postpartum versions) is used after delivery and includes all ten factors of the prenatal version plus three additional risk factors. Total scores on the PDPI-R postpartum version range 0–39 [24]. The higher the score, the more risk factors the subject had for PPD.

### Development of the Japanese version of PDPI-R (PDPI-R-J)

Prior to data collection, we developed the PDPI-R-J. Semantic equivalence focuses on whether the connotative meaning of each item in the translated version of an instrument is identical to that of the original-language version [30]. Back-translation, which is the key to establishing semantic equivalence, was used to assure that the meaning of each item in the PDPI-R remained the same after translation into Japanese.

#### Translation

Two bilingual translators worked independently on translations. One of the translators had a linguistic sociology background, and the other was experienced in broadcasting translations.

#### Committee approach

The two Japanese versions were checked by the first author (MI), and multiple discussions were held with the translators regarding the discrepancies between the two versions; then, one combined version was created. The committee consisted of professionals who convened to discuss the content equivalence and semantic equivalence of the combined version. The members of the committee were two psychologists, a midwife with experience dealing with pregnant women, and two midwives in academic-research fields with experience using this kind of instrument. Any inconsistencies identified were discussed in the committee, comments from the translators were considered, and discussion took place again until consensus was reached.

#### Back-translation

The consensus version was translated back into English by a translator whose native language was English and who was unaware of the original wording of the instrument. The original and back-translated versions were then checked for discrepancies by the researcher and then referred to the original developer of the PDPI-R for discussion. The original developer introduced a few discussion points, which were also addressed. The following content illustrates some of the discussions and decisions.

“Cohabitating” was combined with ‘married’ in the original version to mean “having a stable partner.” However, in Japan, very few couples live together without being officially married. “Cohabitating” usually does not connote a stable relationship—rather, it represents more of a temporary association. Therefore, we decided to exclude the word “cohabitating.”

We paraphrased the term “socioeconomic status” to “*kurashi-muki*,” which means “daily life circumstances,” because the purpose here is to discover the respondents’ perceived financial status. We restated “be comfortably off” as “high” and “narrow circumstances” as “low.”

The original version of the self-esteem item, “feel good about yourself,” was very difficult to translate into Japanese. The Japanese word for “good” has a nuance of evaluation against others instead of against an individual’s own standard. Thus, we phrased it, “Do you like yourself?” to determine whether one likes oneself (i.e., whether or not one is content with oneself, not whether or not others think one is “good”).

The question asked in the postpartum version, “Does your baby cry much?,” was a question often used by the health visitors during home visits to see if the babies were in good health. Therefore, we paraphrased it to “Do you have a hard time soothing the crying baby?”

Assessment questions of social support in the PDPI-R were very particular. The word “confide,” which means to tell a trusted person about personal things, was used. We therefore translated this to “*kokoro-no-uchio-uchiakeru*,” meaning “open your heart” to a trusted person.

We conducted a pretest using the preliminary version and gave it to six mothers for comments. Then, we finalized the Japanese version of the scale.

### Outcome measure: major or minor depression (mMD)

The severity of depressive symptoms was assessed for all participants using the M.I.N.I., which was administered by a trained psychologist. The M.I.N.I. is a short, structured diagnostic interview that was developed jointly by psychiatrists and clinicians in the United States and Europe to detect DSM-IV and ICD-10 psychiatric disorders [31]. With an administration time of approximately 15 minutes, it was designed to meet the need for a short but accurate structured psychiatric interview for multicenter clinical trials and epidemiology studies and to be used as a first step in outcome tracking in non-research clinical settings.

### Statistical analysis

Data are presented as means and percentages. The frequencies of endorsement of dichotomous PDPI-R-J items were compared between the 8^th^ month of pregnancy and the 1^st^ month postpartum using McNemar’s test, while the means of PDPI-R-J items were compared using paired *t*-tests. Logistic analysis was performed in order to establish the relationships between each risk factor of the PDPI-R-J and the occurrence of postpartum mMD. Internal consistency reliability was estimated using Cronbach’s alpha. Factor-to-factor and factor-to-total scale correlations were estimated to determine construct validity. Receiver operating characteristic (ROC) analysis was conducted to determine the cutoffs of the PDPI-R-J scores for the prenatal and postpartum versions. In ROC analysis, sensitivity and specificity are plotted over the range of cutoff points [32]. The area under the curve (AUC) represents the accuracy of the instrument in predicting which women will or will not have PPD. The following interpretation of AUC values is traditional: AUC < 0.7 suggests “low” diagnostic accuracy, AUC 0.7–0.9 “moderate” diagnostic accuracy, and AUC > 0.9 “high” diagnostic accuracy [33]. Analyses were conducted using IBM SPSS Statistics version 20.

### Ethical considerations

Each participant was informed about the purpose of the study both verbally and in writing, and we guaranteed that their information would be treated confidentially. We informed them that their participation in the study was voluntary and that refusal to participate would not disadvantage them. We also informed them that they could withdraw from the research if they wished. The study protocol and the assessment procedures were reviewed and approved by the Ethics Committee of the University of Tokyo.

## Results

### Participant characteristics

Of the 91 who gave their consent to participate, 7 women were hospitalized due to imminent abortion and could not participate in the baseline assessment. Of the 84 enrolled women, 2 did not complete the 1^st^ month postpartum assessment (one was hospitalized due to severe PPD, and the other wished to withdraw from the research). Of the 82 women who completed the 1^st^ month postpartum assessment, 6 were excluded for ineligibility (three were multiparous, two had premature delivery, and one gave birth to an infant with a congenital disorder). The sociodemographic characteristics of the sample are reported in Table 2. The participants’ mean age was 33.4 years (SD = 4.5); 71.1%, 100%, 54%, and 76.8% had a university or graduate degree, were married or had a partner, were employed outside the home, and had medium or high socioeconomic status, respectively. Of the 76 women who completed the 1-month–postpartum assessment, 16 (21%) met the criteria for mMD.

**Table 2:** Characteristics of the participants completed the assessment at one month after delivery (N=76)

**Age, mean (SD)**	**33.4 (4.5)**	
Age range, years	24–43	
Marital status, n (%)		
Married/partnered	76	(100.0)
Single	0	(0.0)
Employment status, n (%)		
Employed	36	(47.4)
Part-time	5	(6.6)
Housewife	35	(46.1)
Educational level, n (%)		
High school	10	(13.2)
Community College	12	(15.8)
University degree	48	(63.2)
Graduate school degree	6	(7.9)
Socioeconomic status, n (%)		
Low	0	(0.0)
Medium	52	(68.4)
High	14	(18.4)

### Feedback from the participants

The follow-up questions were completed by most of the participants. Some referred to their datebooks and diaries to confirm specific facts. Comments made regarding the interviews with the participants included that the topics mentioned in the PDPI-R-J were practical and that they felt at ease talking about their feelings. They also mentioned that writing down their histories of depression made them feel comfortable communicating openly with the researchers.

### Distribution of risk factors

Descriptive statistics for the PDPI-R-J (prenatal and postpartum versions) are reported in Table 3. The most common risk factors in the prenatal version were prenatal anxiety and prenatal depression (85.5% and 23.7%, respectively), and those in the postpartum version were prenatal anxiety, prenatal depression, and maternity blues (82.9%, 17.1%, and 57.9%, respectively). No significant differences in age, employment status, educational level, or socioeconomic status were observed between the depressed and non-depressed groups.

**Table 3:** Risk factors during the 8th months of pregnancy and the 1st month postpartum (N=76)

**PDPI-R-J**		**8th months pregnancy**		**1st month postpartum**		***t *test or McNemar**
Prenatal Items						
F1	Being single, n (%)	0	(0.00)	0	(0.00)	
F2	Low socioeconomic status, n (%)	0	(0.00)	0	(0.00)	
F3	Low self-esteem, mean (SD)	0.51	(0.89)	0.49	(0.83)	*p* = 0.69
F4	Prenatal depression, n (%)	18	(23.7)	13	(17.1)	*p* = 0.18
F5	Prenatal anxiety, n (%)	65	(85.5)	63	(82.9)	*p* = 0.75
F6	Pregnancy intendedness, mean (SD)	0.37	(0.59)	0.36	(0.56)	*p* = 0.82
F7	Prior depression, n (%)	14	(18.4)	11	(14.5)	*p* = 0.45
F8	Lack of social support, mean (SD)	2.29	(1.73)	1.53	(1.7)	*p* < 0.01
F9	Marital dissatisfaction, mean (SD)	0.36	(0.53)	0.39	(0.71)	*p* = 0.63
F10	Life stress, mean (SD)	0.87	(1.02)	0.46	(0.70)	*p* < 0.01
Postpartum items						
F11	Childcare stress, mean (SD)			1.00	(0.8)	
F12	Infant temperament, mean (SD)			1.50	(1.32)	
F13	Maternity blues, n (%)			44	(57.9)	

To determine the relationship between the presence of each risk factor and the likelihood of having postpartum mMD, we used bivariate logistic regression models. The postpartum mMD results at the 1^st^ month postpartum showed that marital dissatisfaction, childcare stress, and infant temperament were associated with having PPD. Table 4 reports the odds ratio (OR) for every risk factor in predicting PPD.

**Table 4:** PDPI-R-J in predicting PPD: bivariate logistic analysis

**PDPI-R-J**		**8th months pregnancy**		**1st month postpartum**	
Prenatal items	OR		(95% CI)	OR	(95% CI)
F1	Being single				
F2	Low socioeconomic status				
F3	Low self-esteem	1.30	(0.73–2.30)	1.54	(0.84–2.83)
F4	Prenatal depression	2.40	(0.73–7.92)	0.64	(0.13–3.21)
F5	Prenatal anxiety	*		*	
F6	Pregnancy intendedness	0.80	(0.29–2.19)	0.83	(0.30–2.35)
F7	Prior depression	1.67	(0.45–6.24)	1.50	(0.35–6.46)
F8	Lack of social support	1.21	(0.88–1.66)	1.30	(0.96–1.76)
F9	Marital dissatisfaction	1.83	(0.69–4.85)	2.13	(1.05–4.34)
F10	Life stress	1.09	(0.64–1.84)	1.70	(0.82–3.52)
Postpartum Items					
F11	Childcare stress			3.39	(1.50–7.68)
F12	Infant temperament			4.04	(1.74–9.37)
F13	Maternity blues			2.63	(0.76–9.07)

*All of those who had prenatal anxiety (F5) met criteria for postpartum depression.

### Reliability of the PDPI-R

Subscale correlation statistics were computed for the PDPI-R. All subscale-to-subscale correlations were either not significant or under 0.491 as shown in Tables 5 and 6. All subscale-to-total correlations were significant (r = 0.25–0.70) except for that of factor 6, unintentional pregnancy. The internal consistency reliability of the PDPI-R was supported by Cronbach’s alpha values of 0.68 for the prenatal version and 0.71 for the postpartum version.

**Table 5:** Spearman’s correlations between factor-to-factor and factor-to-Total of PDPI-R-J prenatal version (N=76)

	**Total scale**	**F3**	**F4**	**F5**	**F6**	**F7**	**F8**	**F9**	**F10**
Total scale	1								
F3	0.588^**^	1							
F4	0.337^**^	*ns*	1						
F5	0.253^*^	*ns*	*ns*	1					
F6	0.404^**^	0.272*	*ns*	*ns*	1				
F7	0.286^*^	*ns*	0.454**	*ns*	*ns*	1			
F8	0.700^**^	0.405**	*ns*	*ns*	*Ns*	*ns*	1		
F9	0.343^**^	*ns*	*ns*	*ns*	0.298**	*ns*	*ns*	1	
F10	0.700^**^	0.241*	0.261*	0.279*	0.296**	*ns*	0.260*	0.272*	1

**p<.01 * p<.05.

**Table 6:** Spearman’s correlations between factor-to-factor and factor-to-Total of PDPI-R-J postpartum version (N=76)

	**Total scale**	**F3**	**F4**	**F5**	**F6**	**F7**	**F8**	**F9**	**F10**	**F11**	**F12**	**F13**
Total scale	1											
F3	0.343^**^	1										
F4	0.276^*^	*ns*	1									
F5	0.503^**^	*ns*	*ns*	1								
F6	0.171	*ns*	*ns*	*ns*	1							
F7	0.250^*^	0.336**	0.310**	*ns*	*ns*	1						
F8	0.660^**^	0.271*	*ns*	0.326**	*ns*	*ns*	1					
F9	0.434^**^	*ns*	*ns*	*ns*	0.257*	*ns*	*ns*	1				
F10	0.517^**^	*ns*	.270*	*ns*	*ns*	*ns*	0.280*	0.491**	1			
F11	0.494^**^	*ns*	*ns*	0.363**	*ns*	*ns*	*ns*	*ns*	*ns*	1		
F12	0.435^**^	*ns*	–.251*	0.234*	−0.260*	*ns*	*ns*	*ns*	*ns*	0.440**	1	
F13	0.324^**^	*ns*	*ns*	0.250*	ns	*ns*	*ns*	*ns*	*ns*	0.226*	*ns*	1

**p<.01 * p<.05.

### ROC analysis

**Figure 1:**
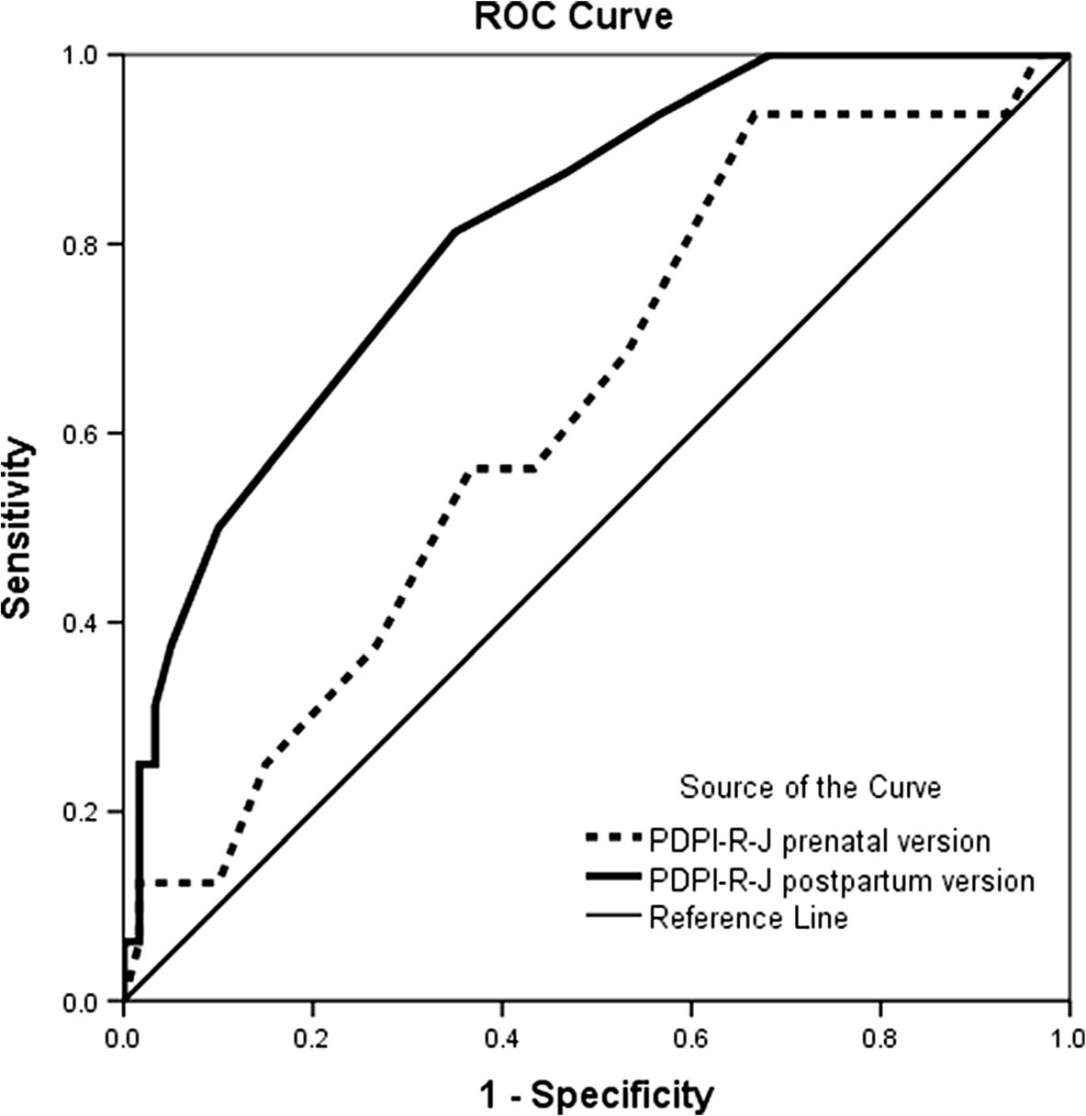
Receiver operating characteristic curve ofPDPI-R-J prenatal version vs. PDPI-R-J postpartum version predicting PPD.

We analyzed the PDPI-R-J as a continuous measure. ROC analyses were performed in order to determine the cutoffs for the prenatal and postpartum versions of the PDPI-R-J. The prenatal version of the PDPI-R-J, which was administered at the 8^th^ month of pregnancy, allowed us to predict 62.8% of PPD cases accurately (Figure 1, Table 5; AUC = 0.628 [95% CI 0.48–0.77]; sensitivity = 0.56 and specificity = 0.57 at a cutoff score of 5.5). For prenatal PDPI-R-J evaluated at baseline, the positive predictive value (PPV), negative predictive value (NPV), and misclassification rate (MR) were 0.28, 0.85, and 42.1%, respectively. The postpartum version of the PDPI-R-J allowed us to predict 82.0% of PPD cases accurately (Figure 1, Table 5; AUC = 0.816 [95% CI 0.71–0.93]; sensitivity = 0.81 and specificity = 0.65 at a cutoff score of 7.5). For the postpartum version of the PDPI-R-J, PPV, NPV, and MR were 0.33, 0.88, and 35.5%, respectively.

**Figure 2:**
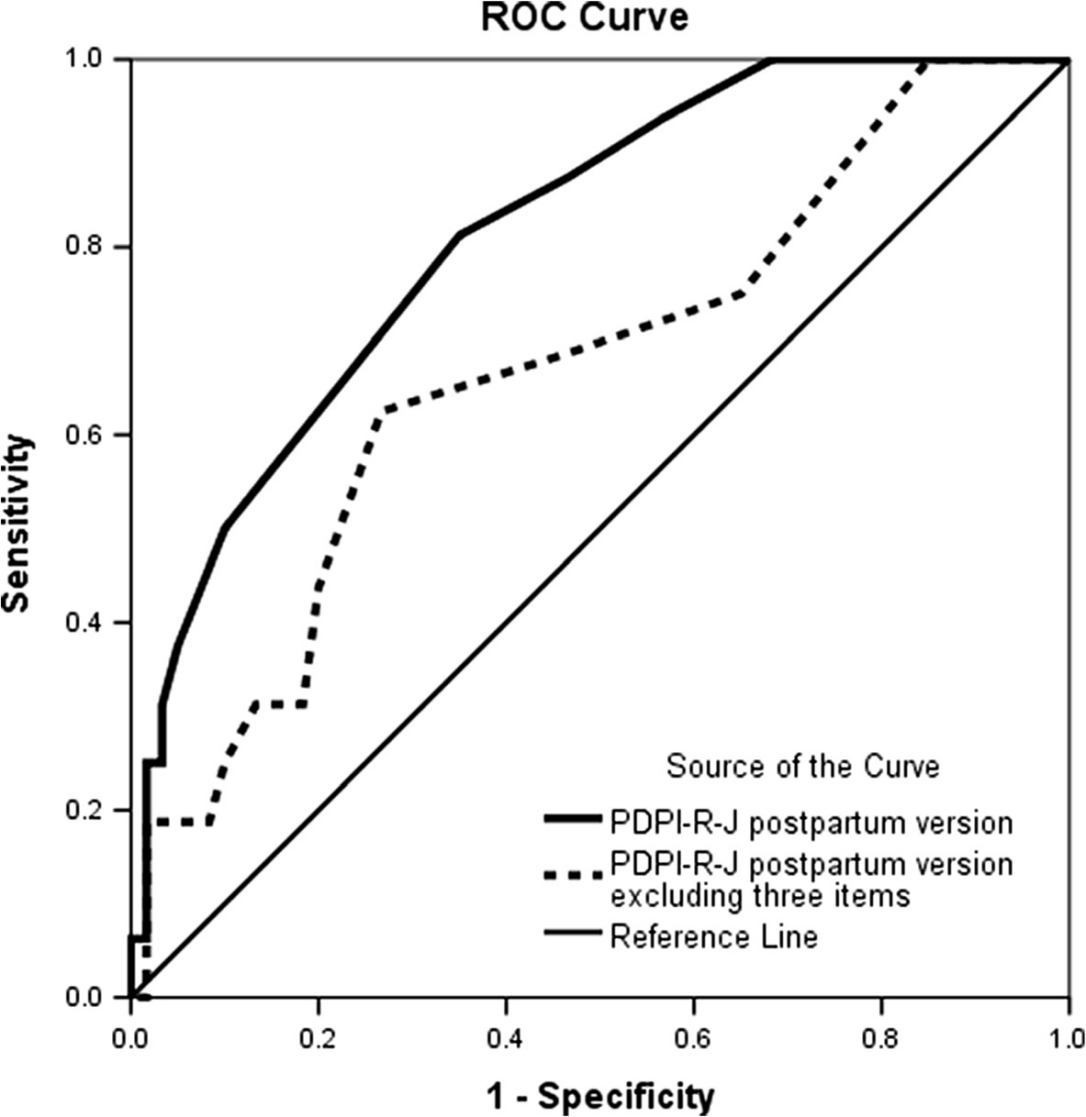
Receiver operating characteristic curve of PDPI-R-J postpartum version vs. PDPI-R-J postpartum version excluding three postpartum items predicting PPD.

A ROC analysis found that the logistic model of the PDPI-R-J postpartum version predicted 82% of PPD cases accurately (Figure 2; AUC = 0.816 [95% CI 0.718–0.93]), whereas the logistic model of the PDPI-R-J postpartum version excluding three postpartum items (#11–13) predicted 67% of PPD cases (Figure 2; AUC = 0.671 [95% CI 0.52–0.82]).

## Discussion

The results of this study suggest that the PDPI-R-J is a useful and easy-to-administer instrument for assessment of risk factors for PPD, in line with the results of the original instrument [24]. Our findings replicate previously published data that emphasize the roles of specific risk factors that occur both during pregnancy and in the postpartum period in predicting PPD. Feedback from the participants indicated that both they and the researcher recognized that their communication was enhanced through the use of the PDPI-R-J. Thus, the opportunity exists to help modify some of these factors before the baby is born. The PDPI-R-J ensures that we engage openly with the women to discuss issues and feelings about which they would not necessarily otherwise speak.

In our study, women reported increased anxiety and life stress and reduced social support during pregnancy, probably due to worry and concern about the imminent delivery. The results suggest that depression during pregnancy and lack of social support are associated with having PPD. These results are consistent with other reports [34,35] that underline that depression during pregnancy and lack of social support usually predict poor adjustment after delivery. The analysis of risk factors conducted at the 1^st^ month after delivery emphasizes the role of childcare stress, infant temperament, low self-esteem, marital dissatisfaction, and other life stressors in predicting the likelihood of PPD. Reliability results were encouraging. The Cronbach’s alpha values obtained were quite satisfactory for a new scale. It may be advisable for future investigations to confirm these results with larger numbers of participants.

The individual risk factors do not allow us to compute cumulative risk (i.e., the overall burden of psychiatric symptoms and distressing life experiences that may increase vulnerability to depression in the perinatal period). Total scores might be more informative than individual items for this purpose. Using the algorithm provided by the original instrument [24], we found that the cutoff score of 5.5 for the prenatal version of the PDPI-R administered at the 8^th^ month of pregnancy had very-similar results regarding sensitivity and specificity. This cutoff is lower than the value of 10.5 provided in the original article but higher than that of 3.5 proposed by Oppo et al. [26]. Cutoff scores can be different for different samples, especially when various cultural backgrounds are considered. Using the postpartum version of the PDPI-R-J, we were able to determine a cutoff score of 7.5 with a sensitivity of 69%.

The prevalence of PPD in our study population was consistent with that identified in previous study [3]. A few women identified as “at risk of developing PPD” were false positives in our study. It is well-known that when the prevalence in the population is low, PPV is less than 50% [36]. There might be controversy surrounding the routine use of screening for depression because of the potential that women “at risk of developing PPD” might not fall under the category. However, it seems that the case for screening outweighs that against it [37,38]. Specifically, screening for perinatal depression is likely to be useful because early intervention may also substantially benefit the woman’s partner and infant [37].

The ROC analysis found that including postpartum items, such as factors related to infant care, increases the predictive validity of mMD. This suggests that women who seemed to be at low risk during pregnancy still need to be tracked continuously in the postpartum period because their risk may depend on their delivery experience and their newborns’ temperament.

The strengths of this study are its longitudinal design, its use of risk-factor assessment according to previously published psychometric properties, and the assessment of mMD using the M.I.N.I., a standardized psychiatric interview.

Several limitations of the study should also be considered. First, participants were recruited from one university hospital in the city area, and both their marital and socioeconomic statuses were uniform. Most of the women who participated had medium or high (86.8%) socioeconomic status, significantly above-average for women in Japan (41.9%) [39]; this could affect the external validity of our results. Second, the number of participants was very small, which could have led to insufficient statistical power to evaluate the effect of the subscale factors to predict PPD and to the predictive validity of the total PDPI-R-J scale. Third, the participants had higher levels of education than the average for Japanese women. The study population might have had a greater awareness of PPD, which could have driven the relatively high prevalence rate. Future research is needed to confirm the predictive validity of this measure in other samples with various characteristics.

Second, the number of participants was very small; this could have led to insufficient statistical power to evaluate differences between predictors of PPD.

## Conclusions

A reliable instrument that can easily identify women with elevated risk of prenatal depression and PPD is the first key step to be taken in primary prevention to reduce the negative impact of this disorder on women, their infants, and their families. The results of our study show that the PDPI-R-J is a valid, feasible instrument for screening for PPD. We recommend the use of the PDPI-R-J in routine clinical practice both during pregnancy and in the postpartum period as a simple screening scale for depression. A total score can be assigned, and clinicians and researchers can evaluate the likelihood that any woman will develop PPD using the proposed cutoff score. Clinicians can also administer this PDPI-R-J to facilitate dialogue with women, as originally proposed for Japanese women [20].

## Competing interests

The authors declare that they have no competing interests.

## Authors’ contributions

MI contributed to the conceptualization and design of the study. MI contacted the original developer of the PDPI-R and took responsibility for the translation process of the instrument. MI carried out data collection and analyses. The manuscript was drafted by MI. Both MI and KK reviewed and contributed to the submitted manuscript. Both authors read and approved the final manuscript.

## Pre-publication history

The pre-publication history for this paper can be accessed here:

http://www.biomedcentral.com/1471-2393/13/112/prepub
